# Investigation of Biomolecule Interactions: Optical-, Electrochemical-, and Acoustic-Based Biosensors

**DOI:** 10.3390/bios13020292

**Published:** 2023-02-18

**Authors:** Ieva Plikusiene, Almira Ramanaviciene

**Affiliations:** 1 NanoTechnas—Center of Nanotechnology and Materials Science, Faculty of Chemistry and Geosciences, Vilnius University, LT-03225 Vilnius, Lithuania; 2 State Research Institute Center for Physical Sciences and Technology, LT-10257 Vilnius, Lithuania

## 1. Background

Today, optical, electrochemical, and acoustic affinity biosensors; immunosensors; and immunoanalytical systems play an important role in the detection and characterization of a number of biological substances, including viral antigens, specific antibodies, and clinically important biomarkers [1,2]. In recent years, advancements in acousto-optic techniques have been made across a variety of medical application fields, particularly in improving the resolution, detection speed, and imaging depth. Theoretical modeling strategies, numerical simulation methods, and recent medical applications have been reviewed [3]. The main advantages of biosensors and immunosensors based on optical and acoustic methods in comparison to other signal transducers are the non-destructive nature of analytical signal registration, real-time measurements, the fast and accurate direct label-free detection of various analytes, and the possibility to perform multiple detections of the analyte with the same surface. Acoustic methods can also provide information about the changes in viscoelastic properties during biosensing layer formation and about the specific interaction with biomolecules. Analytical systems based on electrochemical signal transducers are characterized by high sensitivity, short response time, and low cost. In addition, the impact of non-specific binding of various molecules present in the sample on the registered analytic signal is low; measurements can be performed in turbid, opaque, and colored solutions; the same surface can be analyzed by a few different electrochemical methods after biomolecule interaction; and the dimensions of electrochemical biosensors can be easily reduced, allowing them to be integrated directly into microelectronic devices. They can be suitable for automated detection of a single analyte, or adapted for ultrasensitive multiplexed detection of tumor markers using a disposable immunosensor array [4]. Recently, the practical applications of such analytical systems have expanded significantly from clinical diagnostics to food analysis, quality control of products, environmental studies, and monitoring of industrial processes [5,6]. This can be performed on high molecular weight proteins or low molecular weight molecules.

## 2. Optical Immunosensors and Immunoanalytical Systems

Optical immunosensors and immunoanalytical systems are based on different measurements such as absorbance, index of refraction, scattering, reflectance, photoluminescence, or polarization. The goal of silicon photonic evanescent field biosensors is to bring together the information-rich signal data offered by lab-scale diagnostics at a significantly low cost, with the portability and rapid detection time offered by paper-based assays [7]. Promising immunosensors based on label-free optical methods, such as surface plasmon resonance (SPR) and spectroscopic ellipsometry (SE), can measure changes in the refractive index during biomolecule immobilization or during interactions with an analyte at the solid–liquid interface [8]. SE is extremely sensitive when applied in total internal reflection configuration due to the change in light phase. Additionally, by using SE in total internal reflection mode in combination with SPR, the highest sensitivity is achieved; this method is named total internal reflection ellipsometry (TIRE) [9]. The application of SPR and TIRE for measurements and investigations in real time yields low limits of detection of the analytes of interest, provides the possibility of calculating the affinity of antibodies during their interaction with the antigen, and can follow the formation process of immune complexes. The affinity of an antibody to specific antigens demonstrates how fast and effectively the immune complex is formed. Furthermore, the evaluation of association and dissociation rate constants provides important information about the thermodynamics of such processes [10,11,12,13]. The combination of SE and SPR in TIRE has been successfully applied in the study of the interactions between various mutations (alpha, beta, and gamma) of the SARS-CoV-2 spike protein and specific antibodies present in human blood post-vaccination or after recovery from COVID-19 [9,13,14]. Furthermore, TIRE has been used for the detection of the bovine leukaemia virus antigen gp51 with immunosensors based on a native antibody- or antibody reduced fragment-modified sensing surface. It has also been used for the study of antibody–antigen binding kinetics in real time [9,15]. Additionally, this method was successfully applied in the study of bovine serum albumin (BSA) covalent immobilization on an Al_2_O_3_/ZnO nanolaminate sensing surface and on ZnO nanowires [16,17], and in the study of receptors of granulocyte colony stimulating factor and ligand interactions [11,12,14,18]. Recently, TIRE has attracted a lot of attention for its application in antibody–antigen interaction measurements and immunosensor design, due to its elevated sensitivity compared to SPR [10,13,19,20]. The TIRE method is capable of detecting minuscule changes in the refractive index of the surrounding environment caused by the immobilization of either antigens or antibodies and interactions with the target analyte.

## 3. Acoustic Method for Immunosensing

The acoustic method of quartz crystal microbalance with dissipation (QCM-D) has been applied to measuring the shifts in frequency (ΔF) and energy dissipation (ΔD) of vibrational resonance overtones during protein immobilization and affinity interactions with targeted biomolecules [21]. QCM-D is able to detect changes in surface mass density during the formation of monolayers of biomolecules in real time and to evaluate the viscoelastic properties of such layers due to the measurement of ΔD. Simultaneous measurements of ΔF and ΔD allow one to obtain information about biomolecules orientation on the surface [22,23]. This method has previously been used to study novel coronavirus SARS-CoV-2 nucleocapsid proteins and specific antibody interactions, providing a new opportunity to evaluate antibody flexibility [21]. Additionally, it was shown that parameters registered by QCM-D and plotted as ΔD/ΔF can be successfully applied for the evaluation and distinction between specific antibody fragment interactions with bovine leukaemia virus antigen gp51 and non-specific interactions with BSA [24].

## 4. Electrochemical Biosensors and Immunosensors

Electrochemical biosensors and immunosensors are the most common and widely used for the quantitative detection of various biomolecules and other clinically important analytes. They are very promising and have become a viable alternative to existing laboratory methods. Electrochemical immunosensors have been successfully applied for the direct label-free detection of specific antibodies against SARS-CoV-2 spike protein using electrodes with or without a gold nanostructure [25,26,27]. L-cysteine was incorporated into screen-printed carbon electrodes with electrodeposited gold nanostructures for further covalent immobilization of recombinant SARS-CoV-2 spike proteins (rSpike). Using cyclic voltammetry and differential pulse voltammetry, the affinity interactions of rSpike with specific antibodies were investigated [27]. However, different immunoassay formats and various signal amplification strategies are required for the ultra-sensitive detection of biomolecules. Challenging bioanalytical problems, such as sensitivity and specificity, can be resolved by using various nanoparticles, such as noble metals or metal oxide nanoparticles, as signal amplifying tags [28]. Electrochemical glucose biosensors based on electrochemically synthetized gold nanostructures have been developed, the best enzyme glucose oxidase immobilization methods have been selected, and various glucose detection strategies have been used to develop biosensors that exhibit analytical parameters suitable for glucose detection in human blood or food products [29,30,31]. Therefore, the scientific impact of nanoscience and nanotechnology on the development of sensitive analytical systems is significant [32,33]. One of the novel topics which has recently emerged in the development of sustainable energetic systems is the development of microbial fuel cells based on electro-catalytic processes [34].

This Special Issue of *Biosensors* entitled “**Optical-, Electrochemical- and Acoustic-based Biosensors for the Investigation of Biomolecule Interactions**” is dedicated to all aspects of biosensors and immunosensors regarding the mentioned developments in signal transducers and applications in the direct, label-free or the indirect, ultra-sensitive, and labeled detection of different analytes of interest. It is also dedicated to the evaluation of biomolecule interaction kinetics and to exhibiting the advantages of nanomaterials in practical applications. As an introduction to the present Special Issue, this editorial has summarized some aspects of the development and practical applications of optical, electrochemical, and acoustic biosensors and immunosensors. Original research papers and review articles are welcome.

## Data Availability

Not applicable.
